# CogMamba: Multi-Task Driver Cognitive Load and Physiological Non-Contact Estimation with Multimodal Facial Features

**DOI:** 10.3390/s25185620

**Published:** 2025-09-09

**Authors:** Yicheng Xie, Bin Guo

**Affiliations:** School of Electrical Engineering, Sichuan University, Chengdu 610065, China; 2022141440057@stu.scu.edu.cn

**Keywords:** cognitive load detection, non-contact monitoring, multi-task learning, Mamba, physiological measurement

## Abstract

The cognitive load of drivers directly affects the safety and practicality of advanced driving assistant systems, especially in autonomous driving scenarios where drivers need to quickly take control of the vehicle after performing non-driving-related tasks (NDRTs). However, existing driver cognitive load detection methods have shortcomings such as the inability to deploy invasive detection equipment inside vehicles and limitations to eye movement detection, which restrict their practical application. To achieve more efficient and practical cognitive load detection, this study proposes a multi-task non-contact cognitive load and physiological state estimation model based on RGB video, named CogMamba. The model utilizes multimodal features extracted from facial video and introduces the Mamba architecture to efficiently capture local and global temporal dependencies, thereby further jointly estimating cognitive load, heart rate (HR), and respiratory rate (RR). Experimental results demonstrate that CogMamba exhibits superior performance on two public datasets and shows excellent robustness under the cross-dataset generalization test. This study provides insights for non-contact driver state monitoring in real-world driving scenarios.

## 1. Introduction

According to the report of WHO, road traffic accidents account for 3% to 5% of GDP in most countries [1]. While it is clear that human factors account for a substantial portion of these accidents, the gradual advancement of intelligent driving systems and related assistive technologies holds promise for future improvement [2]. As autonomous driving technology developed, it was classified into five levels by the Society of Automotive Engineers (SAE) [3], but this has not led to a significant decrease in the rate of traffic accidents compared to manual driving by humans [4]. In Level 2 (L2) automation, vehicles can perform basic driving tasks, but continuous driver supervision remains necessary, and the driver must be ready to take control at any moment. In contrast, Level 3 (L3) automation allows the vehicle to handle most driving tasks, although the driver must still intervene in response to take-over requests (TORs). At the same time, since human vehicle operation will gradually decrease, the chances of the driver participating in NDRTs increase. There is a marked difference in cognitive demand when a driver is engaged in an NDRT compared to when they are focused solely on driving [5]. This disparity significantly affects the driver’s ability to resume control of the vehicle swiftly and effectively in unexpected situations [6,7]. Numerous accidents have occurred due to drivers not responding adequately to such transitions [8,9]. Therefore, establishing the Driver Monitoring System (DMS) to monitor driver cognitive load is vital for the continued development of autonomous driving technologies and to improve road traffic safety. At the same time, accurately detecting human cognitive load can effectively help drivers belonging to special groups, such as the hearing-impaired, to better complete autonomous driving tasks [10].

Driver cognitive load detection has attracted growing attention in both academic research and industry applications [11]. Traditional methods typically rely on physiological sensors to monitor the state of the subject [12,13]. Though physiological sensors are effective in monitoring the driver’s state, they also present several limitations. Invasive sensors such as electroencephalography (EEG) [14] are not suitable for vehicle deployment, and non-invasive sensors often require drivers to wear expensive and complex equipment [15], making them impractical for real-world driving scenarios [16]. As a result, contactless methods for the detection of cognitive load have been explored. Some studies have employed non-contact devices (e.g., cameras) to estimate cognitive load [17,18,19]. However, most of them solely focus on eye movement, or utilize non-end-to-end machine learning (ML), which overlooks other physiological responses and the consequent lower generalization.

In fact, HR and RR are critical physiological indicators of cognitive load [20], as fluctuations in cognitive demand often manifest as measurable changes in these signals [21]. To achieve a more comprehensive detection of cognitive load, it is necessary to model both HR and RR in addition to cognitive load [21]. However, training independent models for each task would significantly increase deployment costs and reduce iterative efficiency [22]. Given the intrinsic correlations among cognitive load, HR, and RR, a multi-task model presents a compelling solution [23]. Nevertheless, constructing a vision-based multi-task model for cognitive load detection introduces several challenges. First, integrating multiple tasks with distinct data distributions and learning objectives into a single model increases the complexity of training and optimization compared to single-task models [24]. Second, the relationships among cognitive load, HR, and RR are not consistently stable, making it difficult to infer one metric directly from another through simple joint modeling [25]. Third, vision-based systems often struggle to capture temporal dependencies effectively [26], which also impairs the training efficiency and performance of multi-task models.

For the above reasons, this paper proposes an RGB multi-task video-based driver cognitive load and physiological estimation model (i.e., CogMamba). Building on previous research [27,28,29], the feature extraction module is designed to avoid the computational cost and excessive redundant information by focusing on key facial features of the driver. Moreover, given the importance of physiological indicators such as HR and RR in evaluating driver states, we incorporate remote photoplethysmography (rPPG) to extract blood volume pulse signals from critical facial regions [30,31,32]. We organize these optical signals to construct the multimodal information input, i.e., a spatio-temporal map (STMap) [33,34]. For the feature interaction module, we introduce the Mamba architecture [35]. Aligned feature vectors are passed through stacked Mamba blocks, where the state transition matrix enables continuous temporal updates and efficient dynamic feature enhancement [36]. Leveraging the capabilities of state space models (SSMs), our approach captures long-range dependencies among features, enhancing the model’s ability to infer driver states [35]. Notably, compared to conventional deep learning models like Transformers; with computational complexity $O(N^{2})$, the Mamba structure maintains a linear complexity of O(N), making it more suitable for deployment [37]. This is beneficial for building lightweight models. Following the Mamba module, a lightweight two-layer multilayer perceptron (MLP) is employed for downstream tasks, avoiding more complex structures such as residual convolutions or multi-head attention mechanisms.

In summary, the main contributions of this work are as follows:

- As far as we know, this work is the first end-to-end multi-task non-contact driver cognitive load and physiological estimation model with multimodal facial features from a camera.
- The proposed CogMamba utilizes STMap and key facial features—including landmarks, eye regions, and mouth area—instead of full-frame video input, thereby significantly reducing model parameters and computational load.
- We incorporate the Mamba architecture to enhance the extraction of both local and global temporal features, achieving higher efficiency and lower resource consumption compared to traditional attention mechanisms. This efficiency gain stems from the recursive nature of SSMs, which eliminates the need for attention matrix computation. Additionally, lightweight MLPs are employed to further simplify the model architecture and reduce overall complexity.
- The proposed system demonstrates strong performance in assessing driver cognitive load. Furthermore, the experimental results show that the system performs robustly under varying lighting conditions and across different skin tones.

## 2. Related Works

### 2.1. Contact-Based Cognitive Load Detection

The cognitive load of drivers plays a critical role in their ability to perform driving tasks within autonomous driving environments, underscoring the need for accurate and efficient detection methods [38]. According to the existing literature [39], contact-based cognitive load detection has been widely explored. These traditional approaches typically rely on physiological sensors that must maintain direct contact with the driver’s body to capture signals such as EEG, electrocardiography (ECG), and electrodermal activity (EDA) [40].

For instance, Gerjets et al. developed a model based on EEG signals to evaluate cognitive load [41]. However, due to the inherent challenges in acquiring EEG data, alternative methods using ECG and EDA signals have also been investigated [40]. Despite their effectiveness, these contact-based approaches rely on invasive or semi-invasive sensor configurations, requiring physical contact with the driver’s body [42]. This limitation significantly restricts their applicability in real-world driving scenarios [43].

### 2.2. Non-Contact-Based Cognitive Load Detection

Recent years have seen increasing interest in non-contact cognitive load detection, which eliminates the need for intrusive physiological sensors and is thus suitable for real-world applications such as driving, e-learning, and human–computer interaction. Traditional approaches mainly rely on hand-crafted features derived from visual (e.g., eye tracking, pupil dilation, facial expression) or remote physiological signals (e.g., HR variability from magnetic cardiography, non-contact EEG) [43]. Various machine learning models have been applied for cognitive load classification, including Support Vector Machines (SVMs), Random Forests (RFs), and gradient boosting [44]. For instance, Rahman et al. [17]. employed eye-tracking features and SVM to achieve high accuracy in binary cognitive load classification tasks. Similarly, HRV-based models using classical ML methods such as KNN or Decision Trees have reported high performance when classifying low and high cognitive load [45]. However, these methods often rely heavily on manually selected features and are highly sensitive to environmental noise and subject variability. Moreover, most traditional ML models lack generalization to unseen conditions or participants, which limits their applicability in dynamic and real-world settings.

Deep learning has recently demonstrated superior performance over traditional machine learning across a wide range of perception tasks. Models such as Convolutional Neural Networks (CNNs), Recurrent Neural Networks (RNNs), Vision Transformer (VIT), and their variants (e.g., CNN-LSTM, CNN-Transformer, attention-based networks) [46] have been used to automatically extract and learn representations from raw sensor data, including EEG, eye movement, and video. Notably, some deep learning models have achieved accuracy comparable or superior to handcrafted-feature models. For instance, CNN-based architectures have been applied to gaze heatmaps and achieved high accuracy in classifying task-induced mental workload [17]. Meanwhile, facial expression recognition is of great significance for cognitive load detection. Currently, there are many CNN architectures conducting research in this area, such as AlexNet, VGG16, and ResNet50 [47,48]. Recent efforts also include multimodal deep learning combining video, audio, and physiological data, achieving robust cognitive load estimation through shared representation learning [49]. At the same time, some researchers have attempted to use reinforcement learning methods, such as Bayesian reinforcement learning, to conduct related research on cognitive load [50]. Nevertheless, most of these works treat cognitive load estimation as a single-task problem, often targeting only binary or multi-class classification of load levels. This fails to capture the multifaceted nature of human cognitive loads and limits model utility in complex scenarios where multiple related cognitive and behavioral outputs need to be estimated simultaneously [51].

To address these limitations, we propose a novel approach that leverages multi-task deep learning for non-contact cognitive load detection. Our method is built upon the recently proposed Mamba architecture—a sequence modeling framework that achieves state-of-the-art performance in various vision and language tasks [37]. Mamba introduces SSMs with linear recurrent mechanisms, offering a compelling trade-off between long-range dependency modeling and computational efficiency. We adapt Mamba for the multi-task setting by designing a unified model, and the multi-task formulation improves generalization. To the best of our knowledge, this is the first work to introduce Mamba in the context of cognitive load estimation and the first to formulate a multi-task deep learning solution for non-contact cognitive load assessment.

### 2.3. Mamba

Mamba was initially introduced for efficient long-sequence modeling in natural language processing [37]. With its linear recurrent architecture and selective state updates, Mamba quickly gained traction, leading to multiple variants across domains [52,53]. In vision, Bidirectional State Space Models (BSSMs) were incorporated to form Vision Mamba (Vim) [54], which processes image sequences with enhanced context awareness and positional encoding. Vim achieves faster inference and reduced memory usage compared to Transformers on high-resolution images, making it a strong candidate for visual multi-task learning tasks such as non-contact cognitive load estimation.

## 3. Methodology

### 3.1. Preliminaries

#### 3.1.1. State Space Modeling and Discretization Principles

When dealing with multimodal sequence data, it is crucial to construct an effective model that can describe its dynamic evolution. SSM, a mathematical framework widely used in control systems, has also demonstrated powerful time-series modeling capabilities in deep learning. The basic idea is to use an internal state vector to characterize the dynamic process of input signals over time and generate output responses.

For typical linear time-invariant (LTI) systems in the continuous time domain, a state space model can be represented by the following differential equation:

(1) $$h^{\prime }\left(t\right)=Ah\left(t\right)+Bx\left(t\right),y\left(t\right)=Ch\left(t\right)+Dx\left(t\right)$$

Among them, the memory that records historical inputs is the hidden state of the system $h\left(t\right)\in R^{N}$; the current input is x(t)∈R; and the corresponding output is y(t)∈R. $A\in R^{N\times N}$ is the state transition matrix, which can describe the changing patterns of the state itself; B,C is the projection matrix of the input and state; and *D* is the residual connection term.

In order to adapt to discrete input sequences (such as image frames) in deep learning, the above system must be converted to a discrete-time form. A commonly used method is zero-order hold (ZOH), which assumes that the input remains constant within each sampling period [55]. The parameters of the continuous system can be mapped to the discrete system using matrix exponentials:

(2) $$h_{k}=\bar{A}h_{k-1}+\bar{B}x_{k},y_{k}=Ch_{k}+Dx_{k}$$

Among them, the discretized system matrices are given by $\bar{A}=e^{A\Delta t},\;\bar{B}=\left(\int_{0}^{\Delta t}e^{A\tau }\;d\tau \right)B$, where Δt denotes the sampling time interval. This discretization strategy enables the state space model to be directly integrated into neural networks for processing discrete sequential inputs of arbitrary length, while still preserving the benefits of continuous-time dynamics. In the Mamba architecture, the state transition matrix *A* is not learned as a dense matrix. Instead, it is parameterized as a diagonal matrix with negative real entries via $A=-e^{A_{\log}}$ where $A_{\log}$ is a learnable low-dimensional parameter vector. This formulation guarantees the stability of the system by ensuring all eigenvalues of *A* are negative. To discretize *A*, Mamba applies an efficient element-wise exponential, $\bar{A}=e^{A\Delta t}$, which is computationally inexpensive due to the diagonal form of *A*. The input-dependent nature of Δt further empowers the model to adaptively emphasize or attenuate different parts of the input sequence, thereby enabling selective temporal modeling. This stable, efficient, and input-aware state space formulation is particularly well-suited for processing long-range dependencies in visual sequential data, such as facial dynamics in videos. Our symbols and descriptions are summarized in Table 1.

#### 3.1.2. Advantages of the Convolution Equivalence Form and Mamba Model

Although the above discrete iterative form is very similar to traditional RNNs, further expansion reveals that it is mathematically equivalent to one-dimensional convolution operations. Specifically, if the state evolution of the previous *L* time steps is expanded, a convolution kernel can be constructed by weighting and summing the historical inputs:

(3) $$K=\left[CB,CAB,CA^{2}B,\ldots ,CA^{L-1}B\right]$$

Thus, the output sequence can be obtained by calculating the convolution with the input sequence:

(4) y=x∗K

The advantage of this convolutional form lies in its ability to utilize highly parallelized hardware (such as GPUs) to perform simultaneous computations across the entire sequence, thereby significantly enhancing the efficiency of modeling long sequences.

The Mamba model is developed based on this convolutional representation. It combines a structured state space parameter design with efficient numerical discretization methods, enabling the SSM technique—originally designed for low-speed feedback control—to be extended for application in large-scale visual and signal processing tasks. Compared to attention-based models like Transformers, Mamba does not rely on global attention mechanisms with quadratic complexity. Instead, it maintains good scalability while modeling long-range dependencies through state space recursion.

Additionally, Mamba’s core includes linear projections, gating mechanisms, and nonlinear transformations, enabling it to simulate the linear system behavior of traditional SSMs while also expressing complex nonlinear dynamic processes. This integrated design makes Mamba an ideal foundational architecture for processing multimodal continuous signals, providing a unified dynamic modeling framework for subsequent cross-modal alignment and fusion.

### 3.2. Overview

**Figure 1:**
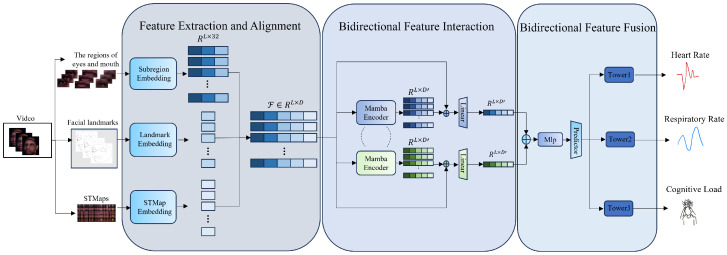
The overall architecture, including feature extraction and alignment, bidirectional feature interaction, and bidirectional feature fusion.

This study develops a non-contact cognitive load detection model for drivers based on Mamba (i.e., CogMamba). The overall method combines the long-time series modeling advantages of the structured state space model Mamba and constructs a multi-stage information processing flow based on task characteristics. As shown in the Figure 1, the overall framework includes the following main stages: feature extraction and alignment, bidirectional feature interaction, bidirectional feature fusion, and optimization target design.

First, to fully capture the dynamic information related to facial regions and physiological signals, the input data undergoes preprocessing and sliding window segmentation, followed by the extraction of local temporal features from multiple key regions. We designed customized feature extraction modules for five parts: left eye, right eye, mouth, facial landmarks, and STMap. We chose them as features for the following reasons. First, the eye and mouth regions are widely recognized as key facial areas that exhibit significant changes under varying cognitive load, such as alterations in blink rate, gaze stability, and mouth movements [56,57]. Second, facial landmarks provide a holistic representation of the entire facial structure and its dynamic states, enabling the capture of subtle changes in head pose, micro-expressions, and overall muscle tension that are also indicative of cognitive load [58,59]. Finally, STMaps are derived from remote photoplethysmography (rPPG) signals and provide temporal patterns of physiological responses, such as heart rate variations, which are closely associated with mental workload [60,61]. By combining these three modalities, our approach leverages both visual and physiological indicators to achieve a more comprehensive and robust estimation of the driver’s cognitive load. Features from different parts vary in expression, temporal characteristics, and spatial density, so they must be aligned using a unified strategy to standardize frame rates and scales after extraction.

After feature alignment, the outputs from the five submodules are serialized into a unified format as input for subsequent temporal modeling. To achieve efficient and structurally aware multi-source information fusion, we introduce the Mamba structure during the feature interaction stage. Specifically, the five serialized feature streams maintain their individual characteristics while undergoing bidirectional modeling through a shared state space dynamic matrix, capturing long-term dependencies and enabling modal-level collaborative dialogue.

Subsequently, the five interaction features processed by the Mamba encoder are further integrated in the bidirectional fusion module. The unified representation after fusion is fed into MLP prediction heads, which output three key physiological and psychological indicators: HR, RR, and cognitive load. Through an end-to-end training mechanism, the model can maintain high temporal resolution while jointly modeling multiple output targets, thereby improving the overall accuracy and robustness of the estimates.

### 3.3. Feature Extraction and Alignment

Given that the structure and consistency of the input have a decisive impact on the effectiveness of the final representation learning, feature extraction and alignment are essential. To fully explore the temporal correlation between local facial regions and physiological indicators, we designed a complete data processing workflow before formally entering the modeling stage, covering the entire chain of operations from raw input organization to high-dimensional feature representation, including data processing, feature extraction, and alignment.

#### Data Processing

Specifically, to ensure temporal consistency, all data segments are sampled using a sliding window method, with a fixed length of L=300 and a step size of s=30 for each sampling, ensuring the continuity and redundancy of local signals on the time axis. This strategy not only improves the stability of the model training process but also lays the foundation for the subsequent establishment of models for long-term dependencies.

**Figure 2:**
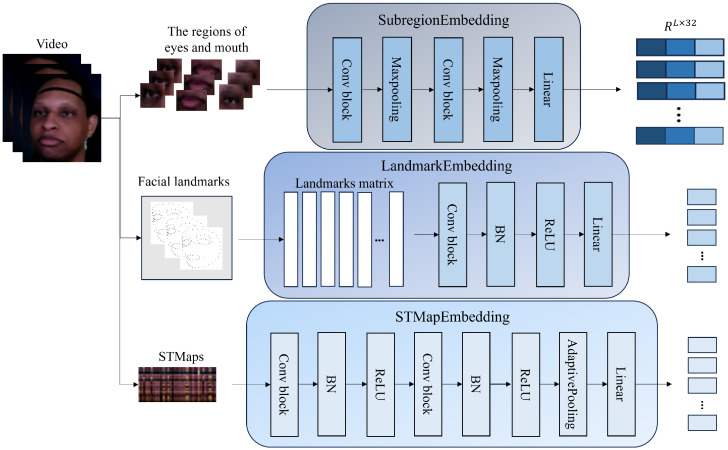
Internal structure of three types of feature embedding.

After sampling, we cropped out local image sequences of the left eye, right eye, and mouth regions. At the same time, we extracted the corresponding facial landmarks’ coordinate information, and an STMap was generated based on rPPG technology for each frame [62,63]. The image sequences of the left eye, right eye, and mouth are unified into a four-dimensional tensor of shape $R^{L\times 3\times H\times W}$, where L=300 denotes the sequence length of 300 frames per segment, and C=3 represents the number of RGB channels. The image sizes vary by region, being 25 × 25 for the eye area and 15 × 35 for the mouth area. In response to this, we designed three customized feature extraction modules (see Figure 2) for five types of input to generate aligned temporal feature vectors. Eye and mouth image sequences are encoded by structurally consistent convolutional networks SubregionEmbedding. This module employs two layers of 2D convolution and pooling operations to extract spatial local features. Prior to convolution, a WTSM module is introduced to apply temporal perturbation to the input, thereby enhancing temporal robustness. After the encoding process, the extracted results are mapped to a fixed-length vector of dimension *D* via a fully connected layer. The output for each region is a sequence feature of shape $R^{L\times d}$.

The facial landmarks are embedded using the LandmarkEmbedding network. This network first reshapes the landmark data into a four-dimensional tensor $R^{L\times 106\times 2}$ and extracts spatial structural features through a convolutional layer with normalization and activation functions. Here, 106 represents the 106 points that constitute the facial landmarks, and 2 represents the two-dimensional plane on which they are located. The extracted features are flattened and input into a fully connected layer, mapped to a *d*-dimensional vector, and then restored to a sequence form $R^{L\times d}$.

The encoding of STMap is performed by the STMapEmbedding module. This module receives a four-dimensional tensor $R^{3\times H\times L}$ as input, which is processed through two layers of convolution, batch normalization, and activation functions. Subsequently, adaptive average pooling is applied to compress the spatial dimension to a uniform width (300 time steps). Finally, the sequence features are permuted and reshaped into $R^{L\times d}$, where the feature dimension is determined by the number of output channels from the convolution layer.

The above five features are strictly aligned in the temporal dimension, as they are all extracted using the same index sliding window method, have consistent lengths, and require no additional interpolation. To achieve a unified representation of information, the five feature sequences are concatenated in the channel dimension to form the final feature sequence $F\in R^{L\times D}$, where D=5∗d. The concatenated features retain the unique structural characteristics of each region while forming a high-dimensional representation with cross-regional semantic complementarity, laying the foundation for a unified representation for subsequent temporal modeling and modal interaction.

### 3.4. Bidirectional Feature Interaction

#### 3.4.1. Feature Extraction and Alignment

**Figure 3:**
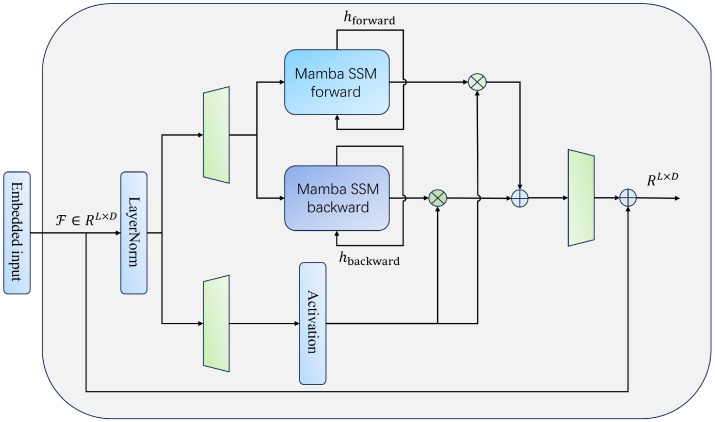
Mamba encoder.

In order to achieve effective coordination and semantic fusion between the temporal features of different facial regions, we introduced the structured state space model Mamba in the modeling stage after feature extraction to construct a bidirectional feature interaction mechanism (Mamba encoder in Figure 3). This module receives the joint feature sequence $F\in R^{L\times D}$ output from the feature extraction and alignment stages, where *L* is the length of the time dimension and *D* is the total feature dimension after concatenation. Considering that the temporal dynamics of different facial regions exhibit bidirectional dependencies—i.e., the physiological state at the current time is influenced by both preceding and subsequent frames—it is necessary to construct a bidirectional sequence modeling structure. We process the input features F through two symmetric Mamba modules for forward and backward modeling, respectively, to obtain dynamic representations in both directions.

Assuming that $F_{\mathrm{forward}}$ and $F_{\mathrm{backward}}$ represent the outputs of the forward and backward encoders, respectively, the Mamba module models them as state space sequence recursions:

(5) $$H_{\mathrm{forward}}={\mathrm{Mamba}}_{\mathrm{forward}}\left(F\right),\;H_{\mathrm{backward}}={\mathrm{Mamba}}_{\mathrm{backward}}\left(F\right)$$

In the specific implementation, the two Mamba modules share the same structure but not the same parameters and take the original sequence and its time-reversed form as inputs, respectively. In each direction, state space modeling essentially performs a series of linear state updates with a recursive structure, the core mechanism of which can be expressed in the following form:

(6) $$h_{t}=Ah_{t-1}+Bx_{t},\;y_{t}=Ch_{t}+Dx_{t}$$

To obtain a unified output dimension, the outputs in both directions are kept consistent in the time dimension, with the same shape as $R^{L\times D^{\prime }}$. Next, $H_{\mathrm{forward}}$ and $H_{\mathrm{backward}}$ are separately input into the linear mapping layer and converted into a unified-dimension embedding representation:

(7) $$Z_{\mathrm{forward}}=\mathrm{Linear}\left(H_{\mathrm{forward}}\right),\;Z_{\mathrm{backward}}=\mathrm{Linear}\left(H_{\mathrm{backward}}\right)$$

The purpose of performing linear mapping is to compress and regularize the temporal dynamic features after interaction, enabling them to retain sufficient information while achieving stronger generalization capabilities. Finally, the outputs from both directions are concatenated to form the fused interaction feature sequence:

(8) $$H_{\mathrm{bi}}=\left[Z_{\mathrm{forward}}\|Z_{\mathrm{backward}}\right]\in R^{L\times 2D^{\prime \prime }}$$

The bidirectional feature interaction module we have created not only enhances the model’s ability to perceive long-term dependent information across different regions of the face but also improves the consistency of cross-modal temporal dynamics through a shared modeling mechanism. Its output serves as input for the subsequent feature fusion stage, continuing the representation construction process in multi-task prediction.

#### 3.4.2. Bidirectional Feature Fusion

Following the bidirectional feature interaction process, the model obtains the feature sequence $H_{\mathrm{bi}}\in R^{L\times 2D^{\prime \prime }}$ output by the Mamba encoder. This sequence simultaneously encodes information from both forward and backward temporal modeling and includes dynamic dependencies among the five categories of facial region features. We have constructed an integrated feature fusion module that combines prediction and fusion to further integrate these bidirectional temporal features and perform multi-task prediction.

The objective of the fusion stage is to compress long-term temporal information and extract high-semantic global representations. Therefore, we first perform pooling operations on $H_{\mathrm{bi}}$ along the temporal dimension. Let the pooling operation be denoted by the function ϕ(·). Typically, average pooling or weighted attention pooling can be selected, but in this study, we adopt average pooling along the temporal dimension to compress the sequence into a fixed-length representation:

(9) $$h_{\mathrm{fused}}=\phi \left(H_{\mathrm{bi}}\right)=\frac{1}{L}\sum_{t=1}^{L}H_{\mathrm{bi}}\left(t\right)\in R^{2D^{\prime \prime }}$$

The global vector $h_{\mathrm{fused}}$ here encapsulates the dynamic information from the entire time period and is transmitted to the task prediction head in a unified feature space.

Upon entering the prediction phase, we constructed a multi-branch MLP module with a shared input, corresponding to the outputs of the three tasks: HR, RR, and cognitive load. Among these, HR and RR are regression tasks, with outputs being scalar real numbers, while cognitive load is a binary classification task, with outputs being probabilities between 0 and 1. Specifically, let the final fused representation be $h_{\mathrm{fused}}\in R^{2D^{\prime \prime }}$. The prediction functions for the three tasks can be formally expressed as

(10) $${\hat{y}}_{\mathrm{hr}}=f_{\mathrm{hr}}\left(h_{\mathrm{fused}}\right),\;{\hat{y}}_{\mathrm{hr}}=f_{\mathrm{hr}}\left(h_{\mathrm{fused}}\right),\;{\hat{y}}_{\mathrm{cog}}=\sigma \left(f_{\mathrm{cog}}\left(h_{\mathrm{fused}}\right)\right)$$

Here, $f_{\;\cdot \;}\left(\;\cdot \;\right)$ represents three independent MLP mappings, and σ(·) is the sigmoid activation function, used to normalize the output to classification probabilities. The entire prediction structure we constructed adopts an end-to-end training approach, with the three tasks sharing the underlying encoder and bidirectional fusion mechanism, branching only at the prediction layer. This structure significantly enhances parameter sharing and information complementarity in multi-task modeling, effectively suppressing task interference while maintaining prediction accuracy. At this point, the model has completed the entire process from raw video segments to multi-task predictions.

### 3.5. Optimization Goal

We have developed differentiated optimization strategies based on the nature of each task’s labels and prediction forms. Within this multi-task learning framework, tasks share a common underlying feature representation structure, while independent loss function branches are set at the top level, enabling joint training through a unified optimization function.

In the cognitive load estimation task, the model must perform a binary classification of the driver’s current load state. Since these labels are primarily derived from subjective questionnaire assessments, their judgment criteria are susceptible to individual subjective differences. Therefore, traditional cross-entropy loss exhibits poor robustness when faced with label bias and inconsistency. To address this, we introduce truncated cross-entropy loss during training to suppress the dominant gradient effect of outlier samples far from the decision boundary during backpropagation, thereby enhancing the model’s adaptability to uncertain labels. Assuming the sample prediction probability is p∈[0,1] and the label is y∈{0,1}, the truncated loss form can be expressed as

(11) $$L_{\mathrm{cog}}=-\log\left(\max(p_{y},\epsilon )\right)$$

where $p_{y}$ is the predicted probability of the true label, and ϵ is the lower threshold to avoid numerical instability, typically set as a small constant.

For the regression task of HR and RR, due to the natural fluctuations in the numerical distribution of physiological indicators, the use of $L_{1}$ loss is sensitive to outliers, which affects model stability. To enhance robustness to marginal samples, we use smooth $L_{1}$ loss as the optimization criterion, which approaches the loss toward linear punishment when the error is large when the error is small, effectively balancing accuracy and stability. For any real-valued prediction $\hat{y}$ and label *y*, the loss is defined as

(12) $$L_{\mathrm{reg}}=\left\{\begin{array}{cc} 0.5{\left(\hat{y}-y\right)}^{2}, & \mathrm{if}\;|\hat{y}-y|<1\\ |\hat{y}-y|-0.5, & \mathrm{otherwise} \end{array}\right.$$

These are used for HR and RR, respectively, denoted as $L_{\mathrm{hr}}$ and $L_{\mathrm{rr}}$.

During the early stages of joint training, specific tasks may introduce unnecessary interference during gradient propagation. Therefore, we introduce a dynamic weight adjustment mechanism to control the multi-task learning for overall optimization. Let the current training iteration be $Iter_{current}$, the total iteration step be $Iter_{total}$, and introduce the time factor *t* and the adaptation weight coefficient λ be

(13) $$t=\frac{{\mathrm{Iter}}_{\mathrm{current}}}{{\mathrm{Iter}}_{\mathrm{total}}}\;\cdot \;2$$

(14) $$\lambda =\frac{2}{1+\exp(-10t)}$$

This design ensures that the model primarily focuses on optimizing the main loss function during the early iteration stages, while the influence of the regularization term gradually increases in later stages. The joint loss expression is as follows:

(15) $$L_{\mathrm{total}}=L_{\mathrm{cog}}+\lambda L_{\mathrm{hr}}+\lambda L_{\mathrm{rr}}$$

Our algorithm 1 is as follows:


**Algorithm 1:** Multimodal facial sequence modeling for HR, RR and cognitive load estimation
**Input**: Video samples with corresponding labels:
            $X^{\mathrm{leye}},X^{\mathrm{reye}},X^{\mathrm{mouth}},X^{\mathrm{facial}},X^{\mathrm{stmap}},y_{\mathrm{hr}},y_{\mathrm{rr}},y_{\mathrm{cog}}$
**Output**: Predicted HR ${\hat{y}}_{\mathrm{hr}}$, RR ${\hat{y}}_{\mathrm{rr}}$, and Cognitive Load ${\hat{y}}_{\mathrm{cog}}$

// Step 1: Feature Extraction

Extract region-specific features: $F_{leye}\leftarrow \mathrm{SubregionEmbedding}\left(X^{\mathrm{leye}}\right)$; $F_{reye}\leftarrow \mathrm{SubregionEmbedding}\left(X^{\mathrm{reye}}\right)$; $F_{mouth}\leftarrow \mathrm{SubregionEmbedding}\left(X^{\mathrm{mouth}}\right)$; $F_{facial}\leftarrow \mathrm{LandmarkEmbedding}\left(X^{\mathrm{facial}}\right)$; $F_{stmap}\leftarrow \mathrm{STMapEmbedding}\left(X^{\mathrm{stmap}}\right)$;

// Step 2: Feature Alignment and Concatenation

Align and concatenate all extracted features along the channel dimension:$F\leftarrow \mathrm{Concat}(F_{leye},F_{reye},F_{mouth},F_{facial},F_{stmap})$;

// Step 3: Bidirectional Feature Interaction via Mamba

$H_{\mathrm{forward}}\leftarrow \mathrm{Mamba}\mathrm{forward}\left(F\right)$; $H_{\mathrm{backward}}\leftarrow \mathrm{Mamba}\mathrm{backward}\left(F\right)$; $Z_{\mathrm{forward}}\leftarrow \mathrm{Linear}\left(H_{\mathrm{forward}}\right)$; $Z_{\mathrm{backward}}\leftarrow \mathrm{Linear}\left(H_{\mathrm{backward}}\right)$; $H_{bi}\leftarrow \mathrm{Concat}\left(Z_{\mathrm{forward}},Z_{\mathrm{backward}}\right)$;

// Step 4: Temporal Aggregation

$h_{fused}\leftarrow \mathrm{MeanPool}\left(H_{bi}\right)$;

// Step 5: Multi-task Prediction

${\hat{y}}_{\mathrm{hr}}\leftarrow f_{hr}\left(h_{fused}\right)$; ${\hat{y}}_{\mathrm{rr}}\leftarrow f_{rr}\left(h_{fused}\right)$; ${\hat{y}}_{\mathrm{cog}}\leftarrow \sigma \left(f_{cog}\left(h_{fused}\right)\right)$;

// Step 6: Compute Loss and Backpropagate

$L_{\mathrm{hr}}\leftarrow \mathrm{SmoothL}1\left({\hat{y}}_{\mathrm{hr}},y_{\mathrm{hr}}\right)$; $L_{\mathrm{rr}}\leftarrow \mathrm{SmoothL}1\left({\hat{y}}_{\mathrm{rr}},y_{\mathrm{rr}}\right)$; $L_{\mathrm{cog}}\leftarrow \mathrm{TruncatedCE}\left({\hat{y}}_{\mathrm{cog}},y_{\mathrm{cog}}\right)$; Compute λ; $L_{\mathrm{total}}\leftarrow L_{\mathrm{hr}}+L_{\mathrm{rr}}+L_{\mathrm{cog}}$; Backpropagate and update parameters.



## 4. Experiment

### 4.1. Datasets and Evaluation Metrics

This study selected two multimodal driving datasets, eDream [64] and MCDD [29], to evaluate the proposed method’s multi-task prediction capabilities for physiological and cognitive loads in natural driving scenarios. The eDream [64] dataset was collected in Canada using the NADS miniSim fixed-base driving simulator and covers 36 participants under the age of 35 (gender-balanced and ethnically balanced). Video was captured using multi-angle cameras (GoPro cameras at 29.97 Hz, Logitech cameras at 30 Hz, and an eye tracker at 60 Hz); physiological signals (ECG and RESP) were sampled at 240 Hz using Becker Meditec sensors, and cognitive load was assessed using the NASA-TLX questionnaire [65]. Scores below 30 were considered low load, and scores above 60 were considered high load.

The MCDD [29] dataset was collected in China (with 42 participants and an average age of 35.28 years (range 23–53 years), primarily of East Asian ethnicity) using the Silab 7.1 system to simulate a driving environment. Video capture was performed using the Orbbec Gemini Pro (640 × 480 resolution, 30 Hz frame rate), and physiological signals were uniformly collected at 100 Hz using Ergoneers devices. Cognitive load was assessed using the NASA-TLX questionnaire, with normalized scores > 10 accompanied by non-driving tasks classified as high load. The dataset included various task settings, with each participant completing 21 driving trials. All video frames were uniformly interpolated to 30 Hz, and physiological signals were synchronously resampled. It is worth noting that both of the datasets we used are publicly available datasets. They were collected after ethical review and approval by the corresponding research teams, and we also signed data sharing agreements with them separately.

Following previous research methods [23,66], we used accuracy, F1 score, sensitivity, and specificity to evaluate the performance of cognitive load estimation. We employed mean absolute error (MAE), root mean square error (RMSE), and Pearson correlation coefficient (P) to assess the model’s performance in predicting HR and RR. Moreover, for each dataset, all subjects were randomly divided into training, validation, and test sets in a 6:2:2 ratio. Model training was conducted on the training set, hyperparameters were tuned using the validation set, and final performance was evaluated exclusively on the test set.

### 4.2. Baselines

To comprehensively evaluate the performance of the proposed CogMamba model in multi-task estimation of cognitive load and physiological parameters, we compared it with several mainstream benchmark methods, including traditional methods, single-task deep learning methods, and multi-task learning models. We reproduced these models based on the source papers of them and tested them using our two datasets to obtain the results.

For cognitive load estimation, we selected several common architectures as single-task models, including CNN [17] based on eye region inputs, LSTM [17], CNN + SVM [17], and AE + SVM [17], representing typical approaches from feature extraction to sequence modeling. Additionally, CLERA [51], a structure specifically designed for eye feature modeling, was also included in the comparison. To analyze the impact of raw video modeling capabilities, we also introduced ResNet3D [67], ViViT [68], and VideoMamba [69] models that take full-face videos as input [33]. VDMoE [29] was the only multi-task model participating in the cognitive load estimation task [23].

In terms of physiological parameter (HR and RR) estimation, we first considered three classic traditional methods: CHROM [70], POS [71], and ARM-RR [72]. These methods are all based on face videos and employ explicit color space transformations or signal processing strategies. Second, four representative single-task HR estimation methods based on deep learning were included: Dual-GAN [73], ConDiff-rPPG [30], HSRD [33], and DG-rPPG [34]. All of these methods use STMap as input and demonstrate varying degrees of performance improvement in HR monitoring. For multi-task estimation of HR and RR, four currently representative deep learning models were selected: MTTS-CAN [74], BigSmall [75], MultiPhys [76], and PhysMLE [22]. The first two use raw video as input, while the latter two adopt the STMap structure, covering various modeling paradigms. VDMoE [29], as a current representative of multi-task fusion structures, provides important references for the comparative analysis of CogMamba through its structural design and loss branch partitioning. Relevant comparison results are detailed in Table 2, Table 3 and Table 4.

### 4.3. Implementation Details

This research model is implemented using the PyTorch (version 2.2.0) framework, and all experiments were conducted on a server equipped with an NVIDIA RTX A6000 graphics card. To extract facial temporal features, facial landmarks are first obtained from the input video and subregion images, and the STMap is constructed based on this to enhance the expression of spatio-temporal information. During the training phase, the Adam optimizer is used, with an initial learning rate of 0.00001, a batch size of 250, and a total number of iterations set to 20,000 to ensure that the model converges adequately under multi-task objectives.

### 4.4. Results of the Comparison Experiment

First, in terms of cognitive load estimation, CogMamba achieved an accuracy rate of 83.56% on the eDream [64] dataset, representing improvements of 28.10% and 25.01% compared to traditional single-task machine learning models such as CNN [17] (65.23%) and LSTM [17] (66.84%), respectively. Compared to the deep learning baseline CLERA [51] (75.82%), there was a 10.21% improvement, and compared to the current representative multi-task model VDMoE [29] (79.89%), there was nearly a 4.6% improvement. Similarly, on the MCDD [29] dataset, CogMamba achieved an accuracy of 81.22%, with superior performance in F1 score (71.62%) and sensitivity (79.33%). These results demonstrate that CogMamba not only possesses stronger feature representation capabilities than traditional machine learning models but also outperforms other deep structures due to the effective modeling of temporal dependencies through the introduction of the Mamba module; Additionally, the multi-task parallel optimization strategy better captures the synergistic associations between cognitive and physiological tasks compared to traditional single-task training.

Furthermore, in terms of physiological signal estimation, CogMamba achieved an HR prediction error (MAE) of 8.97 on the eDream [64] dataset, outperforming all baseline models, with a 24.69% reduction compared to the best multi-task baseline PhysMLE [71] (11.91). In RR prediction, CogMamba’s MAE is 2.20, outperforming PhysMLE [71] (3.22) by approximately 31.68%, while its Pearson correlation coefficient is also the highest currently reported (0.39). On the MCDD [29] dataset, CogMamba continues to lead, with an HR prediction MAE of 10.04, which is 18.36% lower than PhysMLE [71] (12.03). In RR prediction, its MAE is 4.18, which is also 18.7% lower than PhysMLE [71] (5.12).

From a methodological classification perspective, traditional methods (such as CHROM [70] and POS [71]) perform significantly worse in both tasks, with HR prediction errors generally above 17. HR prediction errors for deep learning methods, especially those using STMap input (such as ConDiff-rPPG and HSRD), are reduced to approximately 13–15. However, these methods are designed for a single physiological parameter and lack multi-task generalization capabilities. In contrast, multi-task methods have the ability to learn multiple objectives simultaneously. Among them, CogMamba effectively models long-term temporal dependencies using the Mamba structure, achieving simultaneous improvements in accuracy and efficiency under limited resource constraints.

### 4.5. Results of Ablation Study

We designed multiple ablation experiments to further validate the effectiveness of each submodule in the CogMamba model and compared the results with the complete model, as shown in Table 5 and Table 6. Specifically, we constructed five model variants by removing STMap, facial landmarks, the left eye, the right eye, and the mouth region, respectively. We also compared and analyzed ViViT [68] and ResNet3D [67] as typical video input multi-task model baselines. The experimental results from the eDream [64] and MCDD [29] datasets show that the complete CogMamba achieves the best performance in all three tasks: cognitive load estimation, HR, and RR. In contrast, the removal of each module has varying degrees of impact on performance.

First, when STMap is removed, the model experiences a significant performance decline in physiological signal estimation. On the eDream [64] dataset, the MAE for HR increases from 8.97 to 15.12 (a 68.56% increase), and the MAE for RR increases from 2.20 to 6.41 (a 191.36% increase); on the MCDD [29] dataset, the MAE for HR increases to 16.22, the MAE for RR increases to 8.31, and the *p*-value also decreases significantly. This indicates that the periodic color changes in the facial region captured by STMap are crucial for rPPG modeling, as they explicitly reflect the temporal patterns of heart rate and respiration-related signals [77]. Therefore, relying solely on subregions and facial landmarks is insufficient to recover the fine-grained rhythmic features in physiological signals. Additionally, when the facial landmarks module is removed, the model’s performance degrades across all three tasks, particularly in cognitive load estimation accuracy, which decreases from 83.56% to 81.23%, while the MAE for HR and RR estimation also slightly increases. This change indicates that facial landmarks provide macro-level facial movements (such as frowning or opening the mouth) and expression information, which are highly correlated with cognitive load during driving [58], while also aiding in aligning the structural alignment of the STMap region and enhancing representational consistency.

**Figure 4:**
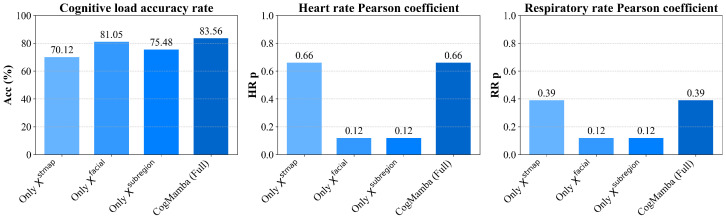
Comparison of test results on the eDream dataset when only one feature is retained.

**Figure 5:**
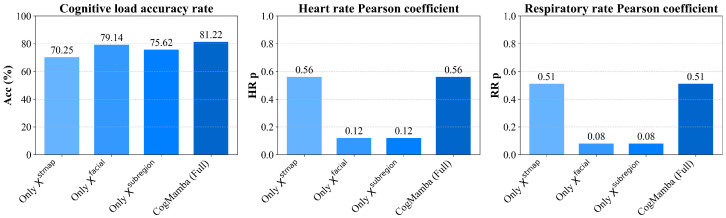
Comparison of test results on the MCDD dataset when only one feature is retained.

Furthermore, after removing the subregion inputs, the performance of cognitive load estimation significantly decreases, with accuracy rates on the eDream [64] dataset dropping to 70.54% and 75.54%, respectively, and F1 scores decreasing by over 4%. This indicates that the eye and mouth regions are critical for cognitive load recognition, particularly the eye region, which is closely related to cognitive load [78]. Additionally, the loss of these regions has a relatively minor impact on HR and RR estimation, suggesting their role is more oriented toward cognitive modeling. At the same time, comparisons of *p*-values across multiple variants show that the complete model maintains the optimal Pearson correlation coefficients for HR and RR (both 0.66 and 0.39), further validating the complementary roles of each submodule in temporal consistency modeling. Thus, each submodule in CogMamba plays a distinct and irreplaceable role. STMap serves as the foundation for physiological modeling; facial landmarks aid in structural alignment and emotional expression; and the eye and mouth subregions enhance the modeling of cognitive cues. We further investigated the performance of the model when only one feature was retained and visualized it as shown in Figure 4 and Figure 5. The integration of these designs enables CogMamba to balance accuracy, robustness, and collaboration across multiple tasks.

### 4.6. Impact of the Number of Mamba Layers

**Figure 6:**
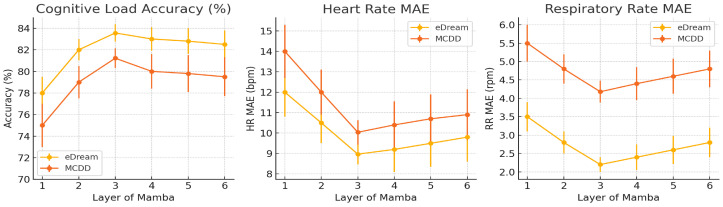
Impact of the number of Mamba layers.

To assess the specific impact of the number of Mamba layers on the performance of the CogMamba model, we systematically tested the performance of Mamba structures with one to six layers in multi-task estimation on the eDream [64] and MCDD [29] datasets. The tasks included cognitive load classification, HR, and RR regression. The experimental results are shown in Figure 6, illustrating the trends in three types of metrics across different numbers of layers.

In terms of cognitive load accuracy, the curves for both datasets show consistent trends: as the number of Mamba layers increases from 1 to 3, accuracy significantly improves, with eDream [64] rising from 78.0% to 83.5% and MCDD [29] from 75.0% to 81.2%, representing increases of 5.5% and 6.2%, respectively. Beyond three layers, accuracy slowly decreases, with the performance at six layers being slightly equivalent to that at two layers. This indicates that stacking an appropriate number of layers can effectively enhance the model’s ability to capture temporal features of driving states, while too many layers may introduce overfitting risks, leading to a decline in generalization performance. For the estimation of HR and RR, both datasets achieved optimal results with a three-layer Mamba structure. In eDream [64], the MAE for HR was 8.97 bpm and the MAE for RR was 2.20 rpm; in MCDD [29], the MAE for HR was 10.04 bpm, and the MAE for RR was 4.18 rpm. The overall trend is similar to that of cognitive load: MAE decreases rapidly between one and three layers, but increases slowly beyond three layers. This phenomenon indicates that a multi-layer Mamba structure helps the model extract more complex temporal–spatial periodic signal features, but excessive stacking may introduce redundant modeling or even noise fitting issues.

This phenomenon can be explained by changes in the learning ability of the sequence modeling mechanism. As the number of layers increases, the Mamba module gains the ability to extract multi-level representations from the original signal, thereby constructing more complex nonlinear function mapping relationships, which facilitates multimodal fusion [79]. However, when stacked too deeply, the model may lose its generalization ability. In addition, too many layers can cause training instability, such as gradient vanishing or explosion [80]. In summary, the three-layer Mamba structure achieves a good balance between accuracy and stability, making it the optimal configuration selected in this study.

### 4.7. Cross-Dataset Estimation

We conducted cross-dataset experiments on the eDream [64] and MCDD [29] datasets to evaluate the generalization ability of the CogMamba model. By training the model on one dataset and testing it on another, we verified the model’s performance under unconstrained conditions in unknown scenarios.

As shown in Table 7, when CogMamba is trained on the eDream [64] dataset and tested on the MCDD [29] dataset, the model maintains good performance across different populations and recording conditions. CogMamba achieves 68.49% accuracy in the cognitive load classification task, outperforming VDMoE [29] (65.97%), ViViT [68] (58.92%), and ResNet3D [67] (57.03%). For HR and RR regression estimates, CogMamba’s HR MAE is 12.66 bpm, slightly higher than VDMoE [29] but significantly better than baselines such as ViViT [68] (16.26 bpm), ResNet3D [67] (17.07 bpm), and MultiPhys [76] (16.26 bpm). The RR MAE is 3.88 rpm, which is not the best but still belongs to the top tier of performance. Similarly, as shown in Table 8, when CogMamba is trained on the MCDD [29] dataset and tested on the eDream [64] dataset, the model maintains its high-quality performance. In the cognitive load classification task, the accuracy reached 66.85%, higher than VDMoE [29] (65.97%), ViViT [68] (56.23%), and ResNet3D [67] (55.43%). The HR MAE was 16.86 bpm, which is at an intermediate level. The RR MAE was 5.72 rpm, slightly higher than VDMoE [29] (5.20 rpm) and DG-rPPG [34] (5.43 rpm), but significantly better than ResNet3D [67] (7.32 rpm) and ViViT [68] (6.93 rpm). Compared to single-task deep models like HSRD [33] and DG-rPPG [34], or multi-task models like PhysMLE [22] and MultiPhys [76] that process STMap inputs, CogMamba generally maintains the same or better performance, demonstrating its strong generalization capabilities.

### 4.8. Results of the Computational Cost Study

Table 9 presents the computational cost comparison in terms of the number of parameters, floating-point operations (FLOPs), and inference time for different models. Among all compared methods, CogMamba achieves the lowest computational overhead, with only 5.95 million parameters, 3.17 GFLOPs, and an inference time of 1.82 ms. This is substantially lower than heavy architectures such as ViViT [68] (87.35 M parameters, 283.06 GFLOPs, 29.81 ms) and ResNet3D [67] (33.37 M parameters, 40.70 GFLOPs, 5.57 ms). Even compared with other lightweight approaches such as VDMoE [29] (7.34 M parameters, 4.05 GFLOPs, 1.91 ms) and HSRD [33] (12.16 M parameters, 4.94 GFLOPs, 1.99 ms), CogMamba still exhibits clear advantages. The combination of a small parameter count, low FLOPs, and short inference time demonstrates the model’s suitability for real-time, resource-constrained deployment scenarios, such as in-vehicle driver monitoring systems, where both efficiency and responsiveness are critical.

### 4.9. Case Study

**Figure 7:**
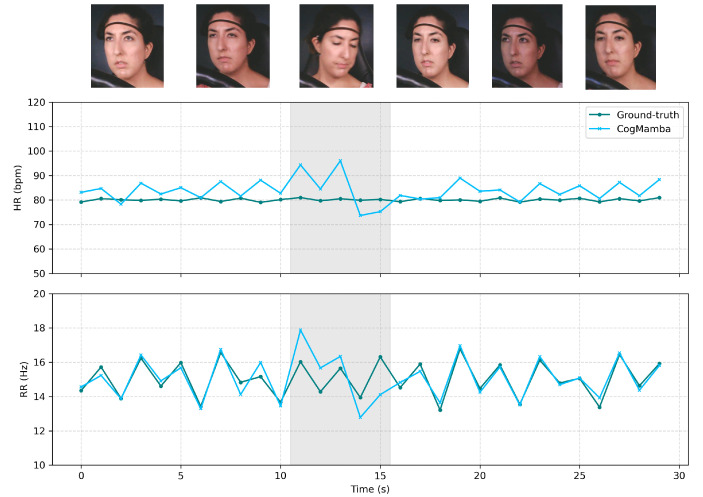
Sampling the impact of facial movement on HR and RR prediction results.

To further investigate the impact of facial movements on the model’s prediction performance, a representative sample from the dataset was selected for testing. As shown in Figure 7, the HR and RR predictions in the gray area exhibit significant deviations when the face is moving and not directly facing the camera, indicating that the model’s ability to extract physiological features decreases during facial movements. This finding highlights a significant challenge in remote cognitive load monitoring—facial actions can impair the accuracy of physiological signal estimation, thus affecting the overall reliability of the system.

## 5. Conclusions

In this study, we propose a multi-task non-contact cognitive load and physiological state estimation model based on RGB video, named CogMamba. Our model combines the advantages of the Mamba architecture while incorporating multimodal facial features through the heterogeneous embedding network. Specifically, compared to traditional deep learning architectures like CNNs and Transformers, our model achieves the ability to capture global context and long-range dependencies while reducing computational complexity, enabling more efficient extraction of shared features. In addition, we extract key regions and facial landmarks from the driver’s face in the video and obtain the driver’s STMap using rPPG technology. By using these three elements as multimodal inputs, the model can focus on truly useful core facial features, thereby reducing the model’s dependence on local non-critical inputs and improving the model’s performance and robustness. To evaluate the feasibility of our method, we tested the model on two public datasets, and the results indicate that CogMamba outperforms other previous models. Therefore, our model has the potential to be deployed in an in-vehicle environment. Meanwhile, since we use multimodal inputs rather than directly inputting video frames into the model, we can effectively protect user privacy. Nevertheless, our model still has room for improvement, particularly in the generalization in cross-dataset testing. In the future, we should train the model on larger datasets and conduct larger-scale generalization tests. Additionally, considering the cost of training the model [32], we need to take into account real-world practical needs and develop more efficient and advanced models.

## Figures and Tables

**Table 1:** Summary of symbols and descriptions.

Symbol	Description
*L*	Length of the input time window (number of frames, e.g., 300)
*D*	Total concatenated feature dimension after alignment
$F_{leye},F_{reye},F_{mouth}$	Feature sequences extracted from left eye, right eye, and mouth
$F_{facial}$	Feature sequence extracted from 106-point facial landmarks
$F_{stmap}$	Feature sequence extracted from STMap (spatio-temporal map)
*F*	Concatenated multi-region feature sequence $\in R^{L\times D}$
$H_{\mathrm{forward}},H_{\mathrm{backward}}$	Forward and backward Mamba-encoded features $\in R^{L\times D^{\prime }}$
$Z_{\mathrm{forward}},Z_{\mathrm{backward}}$	Projected embeddings via linear layers after Mamba
$H_{\mathrm{bi}}$	Concatenated bidirectional features $\in R^{L\times 2D^{\prime \prime }}$
$h_{\mathrm{fused}}$	Temporal-aggregated global representation via mean pooling
${\hat{y}}_{hr},{\hat{y}}_{rr},{\hat{y}}_{cog}$	Predicted heart rate, respiration rate, and cognitive load
$y_{hr},y_{rr},y_{cog}$	Ground truth labels for HR, RR, and cognitive load
$L_{hr},L_{rr}$	Smooth L1 losses for HR and RR regression
$L_{cog}$	Truncated cross-entropy loss for cognitive load classification
λ	Adaptation weight

**Table 2:** Cognitive load estimation performance comparison on the eDream and MCDD datasets.

Method	eDream Dataset					MCDD Dataset			
	Acc (%)	F1 (%)	Sens (%)	Spec (%)		Acc (%)	F1 (%)	Sens (%)	Spec (%)
CNN [17]	65.23	53.18	58.97	71.02		64.01	52.05	58.04	69.56
LSTM [17]	66.84	54.79	60.98	72.71		65.62	53.63	59.95	71.34
CNN + SVM [17]	68.35	56.17	62.94	74.28		67.14	55.02	61.97	72.73
AE + SVM [17]	69.52	57.63	64.48	75.55		68.37	56.41	63.49	74.21
ResNet3D [67]	77.18	65.19	70.96	81.05		72.04	61.10	68.33	73.80
ViViT [68]	78.46	66.97	72.98	82.16		71.76	56.57	57.25	78.63
CLERA [51]	75.82	62.95	68.94	79.53		75.09	62.07	68.02	78.96
VDMoE [29]	79.89	68.96	86.51	77.59		79.96	68.81	77.13	80.77
VideoMamba [69]	80.15	70.25	72.50	84.33		73.00	58.52	58.48	71.11
**CogMamrba**	**83.56**	**74.48**	**88.25**	**82.19**		**81.22**	**71.62**	**79.33**	**84.02**

**Table 3:** Physiological signal estimation performance on the eDream dataset.

Method	Heart Rate (HR)				Respiration Rate (RR)		
	MAE ↓	RMSE ↓	*p* ↑		MAE ↓	RMSE ↓	*p* ↑
CHROM [70]	20.31	25.71	0.21		—	—	—
POS [71]	18.53	22.42	0.25		—	—	—
ARM-RR [72]	—	—	—		4.24	5.11	0.26
Dual-GAN [73]	15.85	18.52	0.47		—	—	—
ConDiff-rPPG [30]	15.41	18.72	0.49		—	—	—
HSRD [33]	14.82	17.91	0.51		—	—	—
DG-rPPG [34]	14.22	17.22	0.53		—	—	—
MTTS-CAN [74]	13.62	16.52	0.57		3.62	4.32	0.31
BigSmall [75]	12.91	15.91	0.58		3.42	4.11	0.32
MultiPhys [76]	12.51	15.41	0.60		3.32	4.01	0.32
PhysMLE [22]	11.91	14.81	0.62		3.22	3.91	0.33
ResNet3D [67]	10.50	14.00	0.65		2.91	3.82	0.37
ViViT [68]	10.21	13.70	0.65		2.82	3.72	0.38
VDMoE [29]	9.17	13.80	0.65		2.53	3.02	0.34
VideoMamba [69]	9.83	**12.72**	0.66		2.45	3.57	0.37
**CogMamba**	**8.97**	13.10	**0.66**		**2.20**	**2.69**	**0.39**

Notes: In this and the following tables, ‘—’ means there are no evaluation results.

**Table 4:** Physiological signal estimation performance on the MCDD dataset.

Method	Heart Rate (HR)				Respiration Rate (RR)		
	MAE ↓	RMSE ↓	*p* ↑		MAE ↓	RMSE ↓	*p* ↑
CHROM [70]	18.33	19.70	0.20		—	—	—
POS [71]	17.33	20.02	0.22		—	—	—
ARM-RR [72]	—	—	—		7.32	9.16	0.11
Dual-GAN [73]	13.29	18.86	0.31		—	—	—
ConDiff-rPPG [30]	15.32	19.58	0.29		—	—	—
HSRD [33]	13.65	17.29	0.30		—	—	—
DG-rPPG [34]	12.97	17.58	0.31		—	—	—
MTTS-CAN [74]	13.96	18.31	0.30		5.22	6.68	0.37
BigSmall [75]	13.13	18.02	0.32		5.26	6.72	0.37
MultiPhys [76]	12.17	18.80	0.45		5.53	7.02	0.38
PhysMLE [22]	12.03	17.11	0.46		5.12	7.03	0.38
ResNet3D [67]	14.27	19.02	0.29		5.63	6.58	0.14
ViViT [68]	14.92	19.61	0.28		5.33	6.10	0.16
VDMoE [29]	10.32	15.37	0.53		4.98	6.53	0.45
VideoMamba [69]	14.02	18.65	0.42		4.53	6.02	0.36
**CogMamba**	**10.04**	**14.20**	**0.56**		**4.18**	**5.39**	**0.51**

**Table 5:** Ablation study on the eDream dataset: cognitive load, HR, and RR estimation.

Model Variant	Cognitive Load			Heart Rate (HR)			Respiration Rate (RR)	
	Acc (%)	F1 (%)		MAE ↓	*p* ↑		MAE ↓	*p* ↑
ResNet3D [67]	77.18	65.19		10.50	0.65		2.91	0.37
ViViT [68]	78.46	66.97		10.21	0.65		2.82	0.38
CogMamba w/o $X^{\mathrm{stmap}}$	82.56	73.65		15.12	0.41		6.41	0.12
CogMamba w/o $X^{\mathrm{facial}}$	81.23	71.23		9.11	0.65		2.61	0.35
CogMamba w/o $X^{\mathrm{leye}}$	70.54	62.01		9.01	0.66		2.34	0.38
CogMamba w/o $X^{\mathrm{reye}}$	70.76	62.23		9.02	0.65		2.35	0.37
CogMamba w/o $X^{\mathrm{mouth}}$	75.54	70.01		8.99	0.66		2.27	0.39
**CogMamba (Full)**	**83.56**	**74.48**		**8.97**	**0.66**		**2.20**	**0.39**

**Table 6:** Ablation study on the MCDD dataset: cognitive load, HR, and RR estimation.

Model Variant	Cognitive Load			Heart Rate (HR)			Respiration Rate (RR)	
	Acc (%)	F1 (%)		MAE ↓	*p* ↑		MAE ↓	*p* ↑
ResNet3D [67]	72.04	61.10		14.27	0.29		5.63	0.14
ViViT [68]	71.76	56.57		14.92	0.28		5.33	0.16
CogMamba w/o $X^{\mathrm{stmap}}$	80.23	70.62		16.22	0.12		8.31	0.08
CogMamba w/o $X^{\mathrm{facial}}$	78.90	70.00		10.11	0.54		4.61	0.50
CogMamba w/o $X^{\mathrm{leye}}$	69.56	60.12		10.11	0.55		4.24	0.50
CogMamba w/o $X^{\mathrm{reye}}$	69.70	60.28		10.12	0.55		4.25	0.50
CogMamba w/o $X^{\mathrm{mouth}}$	75.50	66.91		10.05	0.56		4.27	0.51
**CogMamba (Full)**	**81.22**	**71.62**		**10.04**	**0.56**		**4.18**	**0.51**

**Table 7:** Cross-dataset experiment of training on the eDream and testing on the MCDD dataset.

Method	Cognitive Load			Heart Rate (HR)			Respiration Rate (RR)	
	Acc (%)	F1 (%)		MAE ↓	*p* ↑		MAE ↓	*p* ↑
ResNet3D [67]	57.03	52.63		17.07	0.424		4.96	0.236
ViViT [68]	58.92	51.88		16.26	0.420		4.31	0.226
VDMoE [29]	65.97	60.17		**11.92**	**0.456**		**3.29**	0.238
HSRD [33]	—	—		13.65	0.455		3.79	0.255
DG-rPPG [34]	—	—		13.27	0.455		3.67	**0.264**
MultiPhys [76]	—	—		16.26	0.420		4.31	0.226
PhysMLE [22]	—	—		15.48	0.434		4.18	0.233
**CogMamba**	**68.49**	**62.14**		12.66	0.412		3.88	0.243

**Table 8:** Cross-dataset experiment of training on the MCDD and testing on the eDream dataset.

Method	Cognitive Load			Heart Rate (HR)			Respiration Rate (RR)	
	Acc (%)	F1 (%)		MAE ↓	*p* ↑		MAE ↓	*p* ↑
ResNet3D [67]	55.43	52.77		18.55	0.204		7.32	0.098
ViViT [68]	56.23	59.60		19.40	0.197		6.93	0.113
VDMoE [29]	65.97	58.17		17.75	0.210		**5.20**	0.210
HSRD [33]	—	—		13.42	0.371		6.47	0.315
DG-rPPG [34]	—	—		**13.05**	**0.392**		5.43	**0.357**
MultiPhys [76]	—	—		15.82	0.315		7.19	0.266
PhysMLE [22]	—	—		15.64	0.322		6.66	0.266
**CogMamba**	**66.85**	**60.13**		16.86	0.217		5.72	0.203

**Table 9:** Computational cost comparison of different models.

Model	Param (M)	FLOPs (G)	Inference Time (ms)
ResNet3D [67]	33.37	40.70	5.57
ViViT [68]	87.35	283.06	29.81
VideoMamba [69]	27.65	28.33	4.33
VDMoE [29]	7.34	4.05	1.91
PhysMLE [22]	24.82	34.57	4.96
HSRD [33]	12.16	4.94	1.99
**CogMamba**	**5.95**	**3.17**	**1.82**

## Data Availability

The eDream dataset used in this study can be accessed at https://www.dsp.utoronto.ca/projects/eDREAM/ (accessed on 12 March 2025). For the MCDD dataset, please contact the author of its source paper at jwanggo@connect.ust.hk.

## Abbreviations

The following abbreviations are used in this manuscript:

NDRT	Non-driving-related Task
HR	Heart Rate
RR	Respiratory Rate
SAE	The Society of Automotive Engineers
TORs	Take-over Requests
EEG	Electroencephalography
ML	Machine Learning
rPPG	Remote Photoplethysmography
STMap	Spatial-temporal Map
SSMs	State Space Models
MLP	Multilayer Perceptron
ECG	Electrocardiography
EDA	Electrodermal Activity
SVM	Support Vector Machines
RF	Random Forests
CNN	Convolutional Neural Networks
RNN	Recurrent Neural Networks
BSSM	Bidirectional State Space Models
LTI	Linear Time-invariant
ZOH	Zero-order Hold
MAE	Mean Absolute Error
RMSE	Root Mean Square Error
